# Eficácia e Segurança Terapêutica do Paracetamol versus Ibuprofeno na Persistência do Ducto Arterial em Recém-Nascidos: Uma Revisão Sistemática

**DOI:** 10.36660/abc.20240058

**Published:** 2024-11-13

**Authors:** Halecy Davidson Sousa da Silva, Eryvelton de Souza Franco, Larissa Caroline de Almeida Sousa Lima, Maria Bernadete de Sousa Maia

**Affiliations:** 1 Universidade Federal de Pernambuco Centro de Ciências Médicas Recife PE Brasil Universidade Federal de Pernambuco - Centro de Ciências Médicas, Recife, PE – Brasil; 2 Universidade Federal de Pernambuco Recife PE Brasil Universidade Federal de Pernambuco - Programa de Pós-Graduação em Saúde Translacional, Recife, PE – Brasil; 3 Universidade Federal de Pernambuco Departamento de Fisiologia e Farmacologia Recife PE Brasil Universidade Federal de Pernambuco - Departamento de Fisiologia e Farmacologia, Recife, PE – Brasil

**Keywords:** Persistência do Canal Arterial, Ibuprofeno, Paracetamol, Eficácia, Segurança, Recém-nascidos

## Abstract

O fechamento do canal arterial por meio da utilização de inibidores de cicloxigenase (COX), é considerado o tratamento de primeira linha da persistência do canal arterial hemodinamicamente significativo (PCAHS). Fisiologicamente, as prostaglandinas têm papel reconhecido na PCA (persistência do canal arterial). Reconhecidamente, a eficácia e segurança comparativa entre o ibuprofeno e o paracetamol precisa ser determinada para escolha racional da terapia medicamentosa do fechamento do ducto arterial (DA) em protocolos clínicos.

O presente estudo tem como objetivo apresentar os aspectos da eficácia e da segurança terapêutica do paracetamol versus ibuprofeno no tratamento da PCA em recém-nascidos prematuros.

Procedeu-se a revisão sistemática da literatura, seguindo as recomendações do protocolo PRISMA, utilizando as bases de dados Medline, Pubmed, LILACS e SciELO. Foram incluídos estudos dos últimos 10 anos (2013-2023), os quais analisaram a eficácia e/ou segurança do paracetamol em comparação ao ibuprofeno em recém-nascidos com diagnóstico de PCA.

Foram selecionados para análise 8 ensaios clínicos randomizados (ECRs), resultando em um tamanho amostral de 781 neonatos com PCA tratados com paracetamol ou com ibuprofeno. A eficácia do paracetamol para o fechamento do DA é comparável ao ibuprofeno. Não há diferença estatisticamente significativa na incidência de efeitos adversos relacionadas entre os dois medicamentos na maioria dos estudos.

Há equivalência na eficácia e na segurança do ibuprofeno e paracetamol para promover o fechamento do DA em recém-nascidos prematuros com PCAHS.

**Figure:**
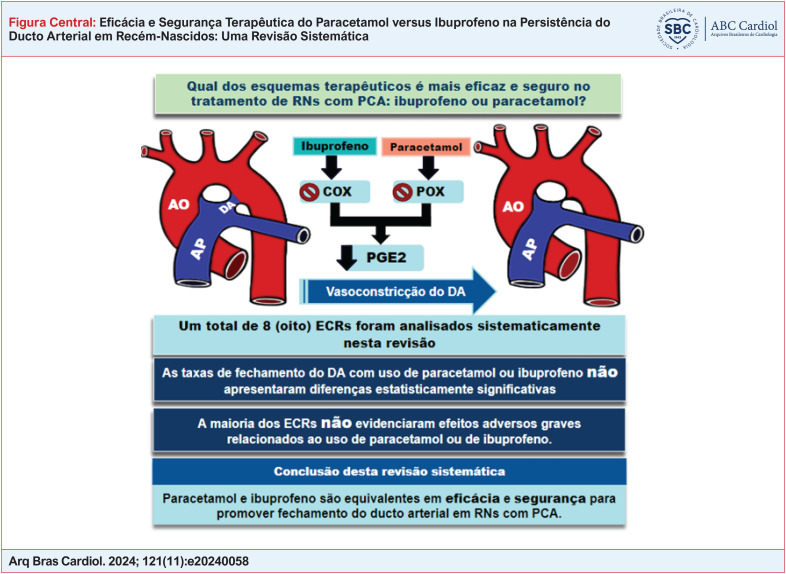
Comparação da eficácia e da segurança do paracetamol em relação ao ibuprofeno no tratamento da PCA em RNs prematuros. DA: ducto arterioso; RNs: recém-nascidos; PCA: persistência do canal arterial; COX: cicloxigenase; POX: peroxidase; PGE2: prostaglandina E2; ECRs: ensaios clínicos randomizados.

## Introdução

O canal arterial conecta a artéria pulmonar e a aorta descendente proximal durante a fase fetal. Apesar da patência do canal ser essencial, o fechamento pós-natal é crucial para a completa independência das circulações sistêmica e pulmonar. Fisiologicamente, o fechamento do ducto arterial (DA) ocorre espontaneamente, em até 72 horas após o nascimento. Todavia, a oclusão pode ser retardada ou inefetiva em recém-nascidos (RN) prematuros, principalmente aqueles com um peso extremamente baixo ao nascer (< 1000g) ou idade gestacional (IG) < 28 semanas.^1^

A persistência do canal arterial hemodinamicamente significativo (PCAHS) é definida e está associada a sinais clínicos ou ecocardiográficos de hipertensão pulmonar e hipoperfusão sistêmica.^2^ Esse é um importante fator de risco para mortalidade e morbidade de RN. Diante disso, o fechamento do canal arterial pode ser feito mediante uso de fármacos inibidores da biossíntese de prostaglandinas na parede da estrutura do canal arterial.

O tratamento da PCA varia desde observação, intervenção medicamentosa ou cirúrgica. Os esquemas terapêuticos utilizados no tratamento da PCA são compostos por inibidores da cicloxigenase (COX), incluindo a indometacina ou o ibuprofeno, e o paracetamol, um inibidor do sítio peroxidase da COX. A terapia padrão para fechamento do PCA, com uso de indometacina ou ibuprofeno, pode causar efeitos adversos como hiperbilirrubinemia, inibição da agregação plaquetária, perfurações gastrointestinais.^3,4^ A escolha da terapia adequada pode ser desafiadora, em razão da necessidade de equilibrar o risco de efeitos adversos e o benefício almejado com a terapia. Embora o ibuprofeno seja a terapia de primeira escolha, seu uso pode oferecer algum risco de lesão renal aguda, proteinúria, sangramento gastrointestinal, perturbação da perfusão cerebral, enterocolite necrosante (ECN) e hemorragia peri-intraventricular (HPIV) em RN prematuros.^5,6^

É presumível que o paracetamol tenha um perfil de segurança superior com potencial de ser uma terapia substituta para os dois medicamentos utilizados atualmente (ibuprofeno e indometacina).^7^ No entanto, ainda são escassos os estudos que avaliaram sistematicamente o perfil de segurança e de eficácia clínica do paracetamol em relação ao ibuprofeno para o fechamento de PCA. O objetivo desta revisão sistemática é apresentar o compilado da literatura direcionada a expor a eficácia e a segurança do paracetamol em relação ao ibuprofeno no fechamento do canal arterial patente no recém-nascido prematuros.

## Métodos

Essa revisão sistemática foi desenvolvida conforme a metodologia *Preferred Reporting items for Systematic Reviews and Meta-analyses* (PRISMA).^8^ Esse estudo respaldou-se nos critérios "PICO", isto é, o acrônimo para População, Intervenção, Controle e Resultados em inglês. A pesquisa foi sistematicamente realizada nas bases de dados PubMed, SciELO, MEDLINE, e LILACS, utilizando os seguintes descritores consultados pelos sites *Medical Subject Headings* (MeSH) e Descritores em Ciência da Saúde (DeCS): *"Efficacy", "Ibuprofen", "Acetaminophen", "Ductus Arteriosus, Patent".* Utilizou-se o operador *booleano* "AND" para agregar os descritores. Objetivou-se avaliar a eficácia e a segurança do paracetamol em relação ao ibuprofeno no tratamento da persistência do canal arterial (PCA) em RN prematuros. Os estudos foram incluídos com base nos seguintes critérios: artigos publicados nos últimos 10 anos (2013 a 2023), com texto disponível na íntegra nos idiomas em inglês e português os quais avaliaram a eficácia e/ou segurança do paracetamol em comparação ao ibuprofeno em RN com diagnóstico de PCA. Foram excluídos estudos duplicados, protocolos clínicos, relatos de casos, estudos de coorte, revisões narrativas, sistemáticas e/ou metanálises, ensaios clínicos que utilizaram tratamento combinado, de braço único, aqueles que avaliaram apenas a eficácia do paracetamol ou do ibuprofeno isoladamente, ou que utilizaram outros AINEs.

### Seleção dos artigos

O processo de triagem foi composto por três etapas. A primeira etapa consistiu na seleção de todos os estudos com os descritores pesquisados no DeCS/MeSH presentes no título, no resumo ou na palavra-chave. Na segunda etapa, os resumos dos estudos foram examinados e os critérios de inclusão e exclusão aplicados. Na terceira etapa, os mesmos critérios estabelecidos foram aplicados também durante a leitura, na íntegra, dos estudos selecionados na etapa de triagem, para avaliar a elegibilidade e, por sua vez, a inclusão dos trabalhos nesta revisão.

## Resultados

### Seleção e características dos estudos

**Figura 1:**
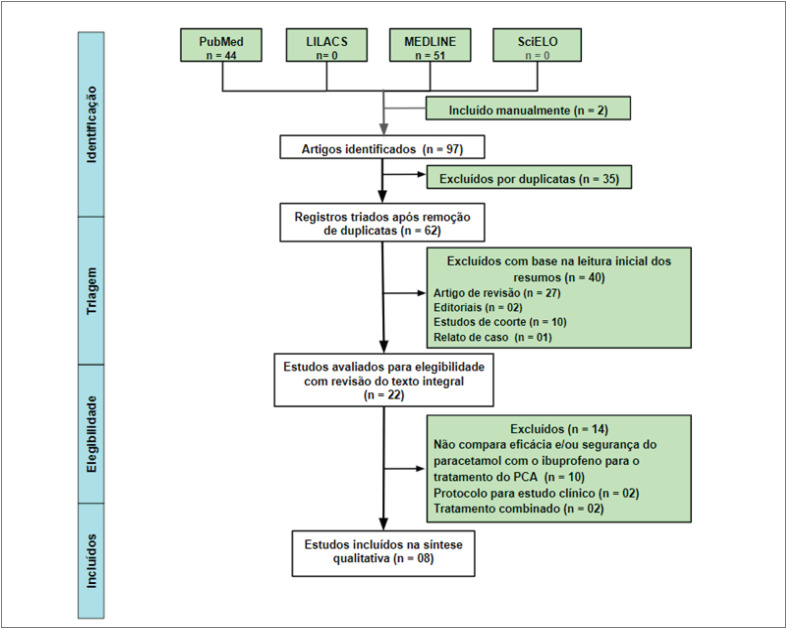
Fluxograma de seleção de artigos.

O fluxograma de seleção de artigos está representado na figura 1. As buscas eletrônicas nas principais bases de dados resultaram em um total de 97 resultados (44 no Pubmed, 0 na LILACs, 51 na MEDLINE e 0 na SCIELO), sendo 2 estudos incluídos manualmente. Foram eliminados 35 artigos duplicados, resultando em 63 estudos. Com base na leitura inicial dos resumos, 40 estudos foram excluídos por serem artigos de revisão (n = 27), editoriais (n = 2), estudos de coorte (n = 9) e relato de caso (n = 1). Após avaliar os 24 artigos elegíveis mediante revisão integral do texto, foram excluídos 14 estudos pelos seguintes motivos: 1) Ensaios clínicos que não compararam eficácia e/ou segurança do paracetamol com o ibuprofeno para o tratamento de PCA (n = 8); 1) protocolo para ensaio clínico (n = 2); 3) Ensaios clínicos que avaliação eficácia do tratamento combinado do paracetamol com ibuprofeno (n = 2). Dessa forma, 8 estudos foram incluídos em síntese qualitativa, resultando em um total de 781 neonatos com PCA tratados com paracetamol ou ibuprofeno. Um resumo dos oito estudos selecionados é apresentado na Tabela 1.

**Tabela 1:** Principais características dos estudos incluídos

Primeiro autor, ano(nº de ref.)	Design do estudo	Critérios de inclusão	Protocolo de tratamento com paracetamol	Protocolo de tratamento com Ibuprofeno	Tamanho da amostra	Principais conclusõessobre a eficácia e a segurança
Al-Lawama et al., 2018^9^	ECR	IG ≤ 32 semanas ou peso ao nascer ≤ 1.500g	10mg/Kg/dose seguido de 1 a 2 mL de solução salina 0,9% a cada 6H por 3 dias	10 mg/kg seguido de 1 a 2 mL de SF 0,9% uma vez ao dia por 3 dias	22 (13/9)	Ibuprofeno oral e o paracetamol são seguros e eficazes no tratamento de PCA
Balachander et al., 2020^10^	ECR	RN prematuros com suspeita clínica de PCA após confirmação ecográfica.	15mg/Kg/dose a cada 6 horas, por 02 dias.	10mg/kg/dose no 1º dia, seguido de 5 mg/kg 24 horas após a primeira dose, por 2 dias.	110 (55/55)	Paracetamol é tão eficaz quando o ibuprofeno no fechamento da PCA, mas a incidência de LRA é maior com ibuprofeno.
Yang et al., 2016^11^	ECR	RN com IG < 37 semanas com até 24 horas de internamento.	15 mg/kg via oral a cada 6 horas por 3 dias.	10 mg/kg seguida de 5 mg/kg durante as primeiras 24 horas e 48 horas depois.	87 (43/44)	A eficácia do ibuprofeno e do paracetamol no tratamento da PCA é semelhante e igualmente segura a curto prazo
Dang et al., 2013^12^	ECR	RN com IG ≤ 34 semanas com PCA confirmação ecográfica.	15 mg/kg 6/6 horas, via oral, por 3 dias.	10 mg/kg seguida de 5 mg/kg, via oral, após 24 horas e 48 horas.	160 (80/80)	Paracetamol tem boa eficácia e menor risco de sangramento gastrointestinal ou hiperbilirrubinemia em comparação com ibuprofeno.
Dani et al., 2021^13^	ECR	IG 25 a 31 semanas e 6 dias com PCA confirmada por ecografia.	15 mg/kg/6 horas, via intravenosa, por 3 dias.	10 mg/kg, seguida de 5 mg/kg após 24 e 48 horas, via intravenosa.	101 (52/49)	Paracetamol é menos eficaz do que o ibuprofeno. O paracetamol é tão seguro quanto o ibuprofeno.
El-Farrash et al., 2019^14^	ECR	IG ≤ 34, idade pós-natal de 2 a 7 dias.	15 mg/kg/6 horas, via oral, por 3 dias.	10 mg/kg/dia seguido de 5 mg/kg/dia, via oral, por mais 3 dias	60 (30/30)	Ibuprofeno e paracetamol são igualmente eficazes e seguros para tratar PCA.
Kumar et al., 2020^15^	ECR	IG < 32 semanas com PCAHS.	15 mg/kg/6 horas, via oral, durante 3 dias.	10 mg/kg/dose, via oral, seguido de 5 mg/kg/dose 24 hs. e 48 hs. após a 1ª dose.	161 (81/80)	Paracetamol oral não é inferior ao ibuprofeno oral no tratamento da PCA.
Oncel et al., 2014^16^	ECR	IG < 32, peso ao nascer < 1250 g e idade pós-natal 48-96 horas e pelo menos um sinal ecocardiográfico de PCA.	15 mg/kg/6 horas durante 3 dias, via oral.	10 mg/kg seguida de 5 mg/kg em 24 e 48 horas.	80 (40/40)	O paracetamol e o ibuprofeno via oral são igualmente eficazes e seguros para o fechamento do PCA.

ECR: ensaio clínico randomizado; IG: idade gestacional; RN: recém-nascido; PCA: persistência do canal arterial.

## Discussão

Os ensaios clínicos analisados nesta revisão evidenciaram que o paracetamol e o ibuprofeno são igualmente eficazes para promover o fechamento do canal arterial, de modo que não houve diferença estatisticamente significativa para ambas as farmacoterapias (Figura Central). Embora a maioria dos estudos tenham demonstrado a segurança terapêutica com paracetamol e com ibuprofeno para o fechamento do DA, alguns ensaios relataram efeitos adversos relacionados ao uso de ibuprofeno, mas sem qualquer repercussão clínica grave.

A maioria dos estudos consideraram como critério de inclusão RNs prematuros, com PCA hemodinamicamente significativa (PCAHS), a partir de critérios ecográficos, tais como diâmetro transductal, reversão do fluxo na aorta descendente, dilatação atrial esquerda; ou presença de sinais clínicos, como precórdio hiperdinâmico, pulsos periféricos limitados e pressão de pulso ampla, choque compensado ou hipotensivo, sinais de subperfusão sistêmica ou superperfusão pulmonar (por exemplo, tempo de enchimento capilar prolongado, diminuição do débito urinário, acidose metabólica, hipotensão, aumento do volume hepático, crepitações pulmonares, edema pulmonar hemorrágico).

Dani et al.,^13^ em recente ensaio clínico evidenciaram que, embora o paracetamol tenha sido menos eficaz no fechamento do PCAHS do que o ibuprofeno (52 vs. 78% p = 0,026), a taxa de sucesso na constricção foi semelhante (81 vs. 90% p = 0,202). Os mesmos autores relatam ainda que o primeiro curso (15 mg/kg/dia por 3 dias) de tratamento com paracetamol foi menos eficaz do que com o ibuprofeno, mas ambos apresentaram efeito de constricção semelhante, visto que estes fármacos não diferem quanto à necessidade de um segundo curso de tratamento, à taxa de reabertura (0 vs. 2%, p = 0,078) e à necessidade de fechamento cirúrgico (0 vs. 2% p = 0,338). O mesmo ensaio reforça que uma segunda administração de ibuprofeno foi eficaz no fechamento do PCA hemodinamicamente significativo e refratário ao primeiro curso de tratamento, sendo assim uma alternativa eficaz e segura ao fechamento cirúrgico.

Por sua vez, Al-Lawama et al.,^9^ em seus estudos demonstraram que a taxa de fechamento primário foi de 69% no grupo paracetamol e 78% no grupo ibuprofeno. Esse mesmo grupo de pesquisadores demonstraram que pela análise do protocolo (62 [95,4%] vs. 63 [94%]; RR 1,01 IC 95% [0,94-1,1]), bem como pelo tempo direcionado ao fechamento do PCAHS não se verificou diferença entre o paracetamol ou ibuprofeno (mediana, 66 horas [IC 95% 61-71 horas] vs. 49 horas [IC 95% 44-54 horas], ambos administrados por via oral. Esses achados corroboram Kumar et al.,^15^ que relatam o fechamento do canal arterial de forma semelhante quando administrado paracetamol ou ibuprofeno via oral.

Ao analisar os estudos desenvolvido por Dang et al.,^12^ verificou-se que o ducto arterial fechou em 81,2% dos lactentes tratados com paracetamol em comparação com 78,8% daqueles tratados com ibuprofeno, porém não houve diferença estatística entre os dois tratamentos (p = 0,693), com intervalo de confiança (IC) de 95% para diferença entre os grupos igual a [-0,080 a 0,128]. Porém, os mesmos autores relatam a possibilidade da reabertura do canal, fato que foi verificado em 05 (cinco) lactentes que fizeram uso de paracetamol e em 06 (seis) lactentes, que fizeram uso de ibuprofeno, mas, com a continuidade do tratamento, o ducto fechou novamente em 4 (quatro) lactentes de cada grupo.

El-Farrash et al.,^14^ verificaram que a taxa de fechamento ductal com o uso de paracetamol oral foi comparável ao ibuprofeno administrado pela mesma via após o primeiro ciclo de tratamento (66,7 vs. 40%, p = 0,272) e após o 2º ciclo de tratamento (80,0% vs. 66,7%, p= 0,929). Ademais, segundo Dang et al.,^12^ mesmo após o primeiro ciclo de tratamento, a diferença do percentual de fechamento ductal não foi estatisticamente significativa (p = 0,268). Assim sendo, a eficácia comparativa não evidenciou inferioridade do paracetamol em relação ao ibuprofeno. Entretanto, Kumar et al.,^15^ reportam que o paracetamol oral pode exigir uma dose mais elevada e/ou um tempo maior para efetuar o fechamento do PCA em relação ao ibuprofeno oral. Apesar de que Oncel et al.,^16^ demonstraram que a taxa de reabertura matematicamente foi maior no grupo do ibuprofeno, entretanto não revelou diferença estatística (24,1% [7 de 29] vs. 16,1% [5 de 31]; p = 0,43).

Quanto aos fatores relacionados a segurança de uso El-Farrash et al.,^14^ relatam que não houve diferença com relação à incidência de efeitos adversos (hemorragia intraventricular, enterocolite necrosante neonatal, displasia broncopulmonar, plaquetopenia, disfunção hepática e renal) e à mortalidade, de forma tal que a incidência desses eventos não foi estatisticamente significativa quando comparado os dois grupos de tratamento. Achados que corroboram Yang et al.,^11^ os quais relataram não ter verificado nenhuma reação adversa relacionada à administração de qualquer um dos fármacos. De forma que os RNs tratados com paracetamol ou ibuprofeno apresentaram resultados semelhantes (p >0,05) para a taxa de fechamento de PCA, sangue oculto nas fezes, incidência de IVH, NEC e DBP.

Nessa mesma perspectiva El-Farrash et al.,^14^ em avaliação de ensaio clínico, verificaram que nenhum dos pacientes desenvolveu ECN, HPIV, sangramento/perfuração gastrointestinal, insuficiência renal/hepática. Ademais Yang et al.,^11^ também não observaram alterações significativas na saturação transcutânea de O^2^, frequência de pulso, pressão arterial, glicemia periférica, bilirrubina transcutânea, temperatura alimentação ou tendência à sangramento, plaquetas, creatinina sérica e transaminase glutâmico-pirúvica e não houve ocorrência de enterocolite necrosante neonatal (ECN). Al-Lawama et al.,^9^ por sua vez, relataram que nenhum dos lactentes apresentou sinais de toxicidade hepática, efeitos colaterais gastrointestinais e não houve diferenças nas complicações neonatais.

Contudo, Balachander et al.,^10^ verificaram a ocorrência de LRA (em todos os estágios) de forma significativamente maior no grupo ibuprofeno (p = 0,024) em relação ao paracetamol. Mas, na maioria dos casos, a LRA foi transitória e os níveis de creatinina retornaram aos valores basais em 48 horas. Vale ressaltar que Dang et al.,^12^ verificaram que a taxa de incidência de sangramento gastrointestinal e hiperbilirrubinemia foi significativamente menor no grupo de RNs com PCA tratado com paracetamol em relação ao grupo que recebeu ibuprofeno (p < 0,05).

Paradoxalmente, os estudos realizados por Balachander et al.,^10^ e por Oncel et al.,^16^ verificaram resultados contraditórios referente ao impacto da terapia medicamentosa no risco de provocar icterícia. Balachander et al.,^10^ revelaram que os neonatos que receberam paracetamol tiveram maior incidência de icterícia, porém sem significância estatística. Por sua vez, Oncel et al.,^16^ verificaram que os níveis de bilirrubina e a função renal e a hepática antes e depois do primeiro e segundo ciclo de paracetamol e ibuprofeno não diferiram significativamente.

## Conclusão

Na maioria dos estudos incluídos nesta revisão sistemática, tanto o ibuprofeno quanto o paracetamol, administrados por via intravenosa ou oral, se mostraram como alternativas igualmente seguras e eficazes no tratamento de PCA em RNs prematuros. Os efeitos adversos verificados tiveram mínimas repercussões clínicas, ao passo que não houve diferenças significativas quanto à eficácia e à segurança quando comparado ambas as terapias medicamentosas. No entanto, alguns estudos evidenciaram menor incidência de LRA, risco de sangramento gastrointestinal e hiperbilirrubinemia com uso o de paracetamol em relação ao ibuprofeno. Diante disso, o paracetamol se apresenta como uma alternativa interessante para o tratamento medicamentoso de PCA em RNs. Não obstante, novos ensaios clínicos multicêntricos são necessários para recrutar um número maior de pacientes, a fim de validar tais resultados em uma amostra populacional maior, mediante aplicação de protocolos mais uniformes.
