# Supplementary material for: Eficácia e Segurança Terapêutica do Paracetamol versus Ibuprofeno na Persistência do Ducto Arterial em Recém-Nascidos: Uma Revisão Sistemática
Source: Arq Bras Cardiol. 2024 Nov 13;121(11):e20240058. [Article in Portuguese] doi: 10.36660/abc.20240058 (PMC11640919; doi:10.36660/abc.20240058)
Supplement: Translation

# Translation: Therapeutic Efficacy and Safety of Paracetamol versus Ibuprofen in Patent Ductus Arteriosus in Newborns: A Systematic Review

**DOI:** 10.36660/abc.20240058i

**Authors:** Halecy Davidson Sousa da Silva, Eryvelton de Souza Franco, Larissa Caroline de Almeida Sousa Lima, Maria Bernadete de Sousa Maia

**Affiliations:** 1 Recife PE Brazil Universidade Federal de Pernambuco - Centro de Ciências Médicas, Recife, PE – Brazil; 2 Recife PE Brazil Universidade Federal de Pernambuco - Programa de Pós-Graduação em Saúde Translacional, Recife, PE – Brazil; 3 Recife PE Brazil Universidade Federal de Pernambuco - Departamento de Fisiologia e Farmacologia, Recife, PE – Brazil

**Keywords:** Patent Ductus Arteriosus, Ibuprofen, Acetaminophen, Efficacy, Safety, Newborn Infant

## Abstract

Closure of the ductus arteriosus (DA) using cyclooxygenase (COX) inhibitors is considered the first-line treatment for hemodynamically significant patent ductus arteriosus (PDA). Physiologically, prostaglandins have a recognized role in PDA. Admittedly, the comparative efficacy and safety between ibuprofen and acetaminophen need to be determined for rational choice of drug therapy for closure of the DA in clinical protocols.

This study aims to present the aspects of the efficacy and therapeutic safety of paracetamol versus ibuprofen in the treatment of PDA in premature newborns.

A systematic review of the literature was carried out, following the recommendations of the PRISMA protocol, using the Medline, Pubmed, LILACS, and SciELO databases. Studies from the last 10 years (2013-2023) that analyzed the efficacy and/or safety of acetaminophen compared to ibuprofen in newborns diagnosed with PDA were included.

Eight randomized clinical trials (RCTs) were selected for analysis, resulting in a sample size of 781 newborns with PDA treated with acetaminophen or ibuprofen. The efficacy of acetaminophen for DA closure is comparable to ibuprofen. There is no statistically significant difference in the incidence of related adverse effects between the two drugs in most studies.

There is equivalence in the efficacy and safety of ibuprofen and acetaminophen to promote closure of the DA in premature newborns with hsPDA.

**Figure:** Comparison of the efficacy and safety of paracetamol in relation to ibuprofen in the treatment of PDA in premature newborns. DA: ductus arteriosus; AO: aorta artery; PA: pulmonary artery NBs: newborns; PDA: patent ductus arteriosus; COX: cyclooxygenase; POX: peroxidase; PGE2: prostaglandin E2; RCTs: randomized clinical trials.

## Introduction

The ductus arteriosus (DA) connects the pulmonary artery and the proximal descending aorta during the fetal phase. Although ductus patency is essential, postnatal closure is crucial for complete independence of systemic and pulmonary circulations.

Physiologically, closure of the DA occurs spontaneously within 72 hours after birth. However, occlusion may be delayed or ineffective in premature newborns (NBs), especially those with extremely low birth weight (< 1000 g)or gestational age (GA) < 28 weeks.^1^

Hemodynamically significant patent ductus arteriosus (PDA) is defined and associated with clinical or echocardiographic signs of pulmonary hypertension and systemic hypoperfusion.^2^ This is an important risk factor for mortality and morbidity in newborns. Therefore, closure of the DA can be achieved by using drugs that inhibit prostaglandin biosynthesis in the wall of the DA structure.

Treatment of PDA ranges from observation to drug or surgical intervention. Therapeutic regimens used in the treatment of PDA consist of cyclooxygenase (COX) inhibitors, including indomethacin or ibuprofen, and paracetamol, an inhibitor of the COX peroxidase site. Standard therapy for PDA closure, using indomethacin or ibuprofen, can cause adverse effects such as hyperbilirubinemia, inhibition of platelet aggregation, and gastrointestinal perforations.^3,4^ Choosing the appropriate therapy can be challenging due to the need to balance the risk of adverse effects and the desired benefit of the treatment. Although ibuprofen is the first-choice therapy, its use may pose some risk of acute kidney injury, proteinuria, gastrointestinal bleeding, cerebral perfusion disturbance, necrotizing enterocolitis (NEC), and peri-intraventricular hemorrhage (PIVH) in premature newborns.^5,6^

It is assumed that paracetamol has a superior safety profile with the potential to be a replacement therapy for the two drugs currently used (ibuprofen and indomethacin).^7^ However, there are still few studies that have systematically evaluated the safety profile and clinical efficacy of paracetamol in relation to ibuprofen for PDA closure. The objective of this systematic review is to present a compilation of the literature aimed at demonstrating the effectiveness and safety of paracetamol in relation to ibuprofen in the closure of the patent DA in premature newborns.

## Methods

This systematic review was developed according to the Preferred Reporting Items for Systematic Reviews and Meta-analyses (PRISMA) methodology.^8^ This study was based on the "PICO" criteria, which is the acronym for Population, Intervention, Control, and Outcomes. The search was systematically carried out in the PUBMED, SCIELO, MEDLINE, and LILACS databases, using the following descriptors consulted by the Medical Subject Headings (MeSH) and Health Science Descriptors (DeCS) websites: "Efficacy", "Ibuprofen", "Paracetamol", "Ductus Arteriosus, Patent". The Boolean operator "AND" was used to aggregate the descriptors. The objective was to evaluate the efficacy and safety of paracetamol in relation to ibuprofen in the treatment of patent ductus arteriosus (PDA) in premature newborns (NB). Studies were included based on the following criteria: articles published in the last 10 years (2013 to 2023), with full text available in English and Portuguese, which evaluated the efficacy and/or safety of paracetamol compared to ibuprofen in newborns diagnosed with PDA. Duplicate studies, clinical protocols, case reports, cohort studies, narrative reviews, systematic reviews and/or meta-analyses, clinical trials that used combined treatment, single-arm trials, those that evaluated only the efficacy of paracetamol or ibuprofen alone, or that used other NSAIDs were excluded.

### Article selection

The screening process consisted of three stages. The first stage consisted of selecting all studies with the descriptors searched in DeCS/MeSH present in the title, abstract, or keyword. In the second stage, the abstracts of the studies were examined, and the inclusion and exclusion criteria were applied. In the third stage, the same criteria established were also used during the full reading of the studies selected in the screening stage to assess eligibility and, in turn, the inclusion of the works in this review.

## Results

### Selection and characteristics of the studies

The article selection flowchart is shown in Figure 1. Electronic searches in the main databases resulted in a total of 97 results (44 in Pubmed, 0 in LILACs, 51 in MEDLINE, and 0 in SCIELO), with 02 studies being manually included. Thirty-five duplicate articles were eliminated, resulting in 63 studies. Based on the initial reading of the abstracts, 40 studies were excluded because they were review articles (n = 27), editorials (n = 02), cohort studies (n = 09), and case reports (n = 01). After evaluating the 24 eligible articles through full-text review, 14 studies were excluded for the following reasons: 1) Clinical trials that did not compare the efficacy and/or safety of paracetamol with ibuprofen for the treatment of PDA (n = 08); 1) clinical trial protocol (n = 02); 3) Clinical trials evaluating the efficacy of combined treatment with paracetamol and ibuprofen (n = 02). Thus, 08 studies were included in qualitative synthesis, resulting in a total of 781 neonates with PDA treated with paracetamol or ibuprofen. A summary of the eight selected studies is presented in Table 1.

**Figure 1:** Article selection flowchart.

**Table 1:** Main characteristics of the included studies

First author, year(ref. nº.)	Study design	Inclusion criteria	Paracetamol tratment protocol	Ibuprofen tratment protocol	Sample Size	Key Findings on Efficacy and Safety
AL-LAWAMA, 2018^9^	RCT	GA *≤* 32 weeks or birth weight *≤* 1.500g	10mg/Kg/dose followed by 1 to 2 mL of 0.9% saline solution Every 6 hours for days.	10 mg/kg followed by 1 to 2mL of 0,9% saline solution once daily for 3 days	22 (13/9)	Oral ibuprofen and paracetamol are safe and effective in treating PDA
Balachander, 2020^10^	RCT	NB premature with clinical suspicion of PDA after ultrasound confirmation.	15mg/Kg/dose every 6 hours for 02 days.	10mg/kg/dose on the 1st day, followed by 5 mg/kg 24 hours after the first dose, for 2 days.	110 (55/55)	Paracetamol is as effective as ibuprofen in closing PDA, but the incidence of AKI é higher with ibuprofen.
YANG, 2016^11^	RCT	NB with GA < 37 weeks with up to 24 hours of hospitalization.	15 mg/kg orally every 6 hours for 3 days.	10 mg/kg followed by 5 mg/kg during the first 24 and 48 hours later.	87 (43/44)	The efficacy of ibuprofen and paracetamol in the treatment of PDA is similar and equally safe in the short term.
DANG, 2013^12^	RCT	NB with GA ≤ 34 weeks with PDA ultrasound confirmation.	15 mg/kg orally every 6 hours for 3 days.	10 mg/kg followed by 5 mg/kg orally after 24 and 48 hours.	160 (80/80)	Paracetamol has good efficacy and a lower risk of gastrointestinal bleeding or hyperbilirubinemia compared to ibuprofen.
DANI, 2021^13^	RCT	GA 25 to 31 weeks and 6 days with PDA ultrasound confirmation.	15 mg/kg/6 hours, intravenously, for 3 days.	10 mg/kg, followed by 5 mg/kg after 24 and 48 hours, intravenously.	101 (52/49)	Paracetamol is less effective than ibuprofen. Acetaminophen is as safe as ibuprofen.
EL-FARRASH, 2019^14^	RCT	GA ≤ 34, postnatal age 2 a 7 days.	15 mg/kg/6 hours, orally, for 3 days.	10mg/kg/day followed by 5 mg/kg/day orally for another 3 days	60 (30/30)	Ibuprofen and paracetamol are equally effective and safe for treating PDA.
KUMAR, 2020^15^	RCT	GA < 32 weeks with hsPDA.	15 mg/kg/6 hours, orally, for 3 days.	10 mg/kg/dose orally, followed by 5 mg/kg/dose 24 and 48 hours after the 1st dose.	161 (81/80)	Oral paracetamol is not inferior to oral ibuprofen in the treatment of PDA.
ONCEL, 2014^16^	RCT	GA < 32, birth weight < 1250g postnatal age 48-96 hours, and at least one echocardiographic sign of PDA.	15 mg/kg/6 hours for 3 days, orally.	10 mg/kg followed by 5 mg/kg in 24 and 48 hours.	80 (40/40)	Oral paracetamol and ibuprofen equally effective and safe for PDA clorure.

RCT: randomized clinical trials; GA: gestational age; NB: newborn; PDA: patent ductus arteriosus.

## Discussion

The clinical trials analyzed in this review showed that paracetamol and ibuprofen are equally effective in promoting closure of the DA so there was no statistically significant difference for both pharmacotherapies (Central Illustration). Although most studies demonstrated the therapeutic safety of paracetamol and ibuprofen for closure of the DA, some trials reported adverse effects related to the use of ibuprofen, but without any serious clinical repercussions.

Most studies considered as inclusion criteria premature newborns (NBs) with hemodynamically significant PDA (hsPDA), based on ultrasound criteria, such as transductal diameter, reversal of flow in the descending aorta, left atrial dilation, or presence of clinical signs, such as hyperdynamic precordium, limited peripheral pulses, and wide pulse pressure, compensated or hypotensive shock, signs of systemic underperfusion or pulmonary overperfusion (for example, prolonged capillary filling time, decreased urine output, metabolic acidosis, hypotension, increased liver volume, pulmonary crackles, hemorrhagic pulmonary edema).

Dani et al.,^13^ in a recent clinical trial, showed that, although paracetamol was less effective in closing the hsPDA than ibuprofen (52 vs. 78% p = 0.026), the success rate in constriction was similar (81 vs. 90% p = 0.202). The same authors also reported that the first course (15 mg/kg/day for 3 days) of treatment with paracetamol was less effective than with ibuprofen, but both had a similar constriction effect since these drugs did not differ in the need for a second course of treatment, the reopening rate (0 vs. 2%, p = 0.078) and the need for surgical closure (0 vs. 2% p = 0.338). The same trial reinforces that a second administration of ibuprofen was effective in closing a hemodynamically significant PDA that was refractory to the first course of treatment, thus being an effective and safe alternative to surgical closure.

In turn, Al-Lawama et al.,^9^ in their studies, demonstrated that the primary closure rate was 69% in the paracetamol group and 78% in the ibuprofen group. This same group of researchers showed that through the analysis of the protocol (62 [95.4%] vs. 63 [94%]; RR 1.01 95% CI [0.94-1.1]),as well as the time directed to the closure of the hsPDA, no difference was observed between paracetamol or ibuprofen (median, 66 hours [95% CI 61-71 hours] vs. 49 hours [95% CI 44-54 hours], both administered orally. These findings corroborate Kumar et al.,^15^ who reported similar closure of the DA when paracetamol or ibuprofen was administered orally.

When analyzing the studies developed by Dang et al.,^12^ it was found that the DA closed in 81.2% of infants treated with paracetamol compared to 78.8% of those treated with ibuprofen, but there was no statistical difference between the two treatments (p = 0.693), with a 95% confidence interval (CI) for the difference between the groups equal to [-0.080 to 0.128]. However, the same authors report the possibility of reopening the ductus, a fact that was verified in 05 (five) infants who used paracetamol and in 06 (six) infants who used ibuprofen, but with the continuation of the treatment, the ductus closed again in 4 (four) infants in each group.

El-Farrash et al.,^14^ found that the ductal closure rate with the use of oral paracetamol was comparable to ibuprofen administered by the same route after the first treatment cycle (66.7 vs. 40%, p = 0.272) and after the 2nd treatment cycle (80.0% vs. 66.7%, p = 0.929). Furthermore, according to Dang et al.,^12^ even after the first treatment cycle, the difference in the percentage of ductal closure was not statistically significant(p = 0.268). Therefore, the comparative efficacy did not show the inferiority of paracetamol in relation to ibuprofen. However, Kumar et al.,^15^ reported that oral paracetamol may require a higher dose and/or a longer time to effect PDA closure in relation to oral ibuprofen. Although Oncel et al.,^16^ demonstrated that the reopening rate was mathematically higher in the ibuprofen group, however, it did not reveal a statistical difference (24.1% [7 of 29] vs. 16.1% [5 of 31]; p = 0.43).

Regarding factors related to the safety of use, El-Farrash et al.,^14^ reported that there was no difference regarding the incidence of adverse effects (intraventricular hemorrhage, neonatal necrotizing enterocolitis, bronchopulmonary dysplasia, thrombocytopenia, liver and kidney dysfunction) and mortality so that the incidence of these events was not statistically significant when comparing the two treatment groups. These findings corroborate those of Yang et al.,^11^ who reported not having observed any adverse reactions related to the administration of any of the drugs. Thus, newborns treated with paracetamol or ibuprofen presented similar results (p>0.05) for the rate of PDA closure, fecal occult blood, and the incidence of IVH, NEC, and BPD.

In the same perspective, El-Farrash et al.,^14^ in a clinical trial evaluation, found that none of the patients developed NEC, PIVH, gastrointestinal bleeding/perforation, or renal/hepatic failure. Furthermore, Yang et al.,^11^ also did not observe significant changes in transcutaneous O2 saturation, pulse rate, blood pressure, peripheral blood glucose, transcutaneous bilirubin, feeding temperature or bleeding tendency, platelets, serum creatinine, glutamic-pyruvic transaminase, and there was no occurrence of neonatal necrotizing enterocolitis (NEC). Al-Lawama et al.,^9^ in turn, reported that none of the infants showed signs of liver toxicity or gastrointestinal side effects, and there were no differences in neonatal complications.

However, Balachander et al.,^10^ found that AKI (at all stages) was significantly higher in the ibuprofen group (p = 0.024) compared to paracetamol. However, in most cases, AKI was transient, and creatinine levels returned to baseline values within 48 hours. It is worth mentioning that Dang et al.,^12^ found that the incidence rate of gastrointestinal bleeding and hyperbilirubinemia was significantly lower in the group of newborns with PDA treated with paracetamol compared to the group that received ibuprofen (p < 0.05).

Paradoxically, the studies carried out by Balachander et al.,^10^ and Oncel et al.,^16^ found contradictory results regarding the impact of drug therapy on the risk of causing jaundice. Balachander et al.,^10^ revealed that newborns who received paracetamol had a higher incidence of jaundice, although without statistical significance. In turn, Oncel et al.,^16^ found that bilirubin levels and renal and hepatic function before and after the first and second cycles of paracetamol and ibuprofen did not differ significantly.

## Conclusion

In most studies included in this systematic review, both ibuprofen and paracetamol, administered intravenously or orally, were shown to be equally safe and effective alternatives for the treatment of PDA in premature newborns. The adverse effects observed had minimal clinical repercussions, while there were no significant differences in efficacy and safety when comparing both drug therapies. However, some studies showed a lower incidence of AKI, risk of gastrointestinal bleeding, and hyperbilirubinemia with the use of paracetamol compared to ibuprofen. Therefore, paracetamol appears to be an interesting alternative for the drug treatment of PDA in newborns. Nevertheless, new multicenter clinical trials are needed to recruit a larger number of patients in order to validate these results in a larger population sample by applying more uniform protocols.
